# Not Always ‘Slapped Cheeks’: Parvovirus B19 Infection Causing Severe Anaemia in a Kidney Transplant Recipient

**DOI:** 10.1155/crit/4150465

**Published:** 2026-08-19

**Authors:** Abdullahi Abdisalan, Uko Otoabasi, David Makanjuola, John Clark, Mysore K. Phanish

**Affiliations:** ^1^ Renal and Transplantation Unit, St Helier Hospital, Epsom and St Helier University Hospitals NHS Trust, London, UK, nhs.uk; ^2^ City St Georges University of London, London, UK

## Abstract

Infections are a significant cause of morbidity and mortality in kidney transplant patients. The clinical presentation can be atypical, and establishing the diagnosis in a timely manner can be challenging. We describe a 55‐year‐old woman who received a kidney transplant and developed severe anaemia with very high ferritin levels, abnormal liver function tests and no evidence of bleeding. Initial screening for parvovirus infection with IgG and IgM antibodies was negative, but blood DNA polymerase chain reaction (PCR) for parvovirus was strongly positive with a high viral load. Treatment with reduction in immunosuppressive therapy and a course of intravenous immunoglobulin resulted in sustained improvement in haemoglobin levels and a reduction in viral load. This case report highlights the diagnostic and management challenges of parvovirus infection in a kidney transplant recipient.

**Unique Features of This Case and Key Learning Points**

1. Parvovirus B19 infection can cause severe anaemia in kidney transplant recipients, and early whole blood PCR to detect the virus is essential to make a diagnosis even in the absence of supportive serology.
2. As in our case, serology can be negative (and seroconversion can be delayed) in patients on immunosuppression leading to a delay in diagnosis.
3. Although self‐limiting in an immunocompetent host, as in our case, viraemia can persist for several months with a risk of symptomatic anaemia during periods of increased immunosuppression.

## 1. Introduction

Infections are a significant risk in solid organ transplant recipients due to immunosuppressive therapy, with viral pathogens such as parvovirus B19 posing unique challenges [1]. Although parvovirus B19 is commonly encountered in childhood, it can cause severe haematological complications in immunocompromised adults [2, 3]. This case report describes a kidney transplant recipient who developed severe symptomatic anaemia due to parvovirus B19 infection. We highlight atypical features in her presentation and laboratory findings, diagnostic challenges and a successful approach to management for treatment of the virus and preservation of renal allograft function.

## 2. Case

We present a clinical case of a 55‐year‐old female with end‐stage renal failure secondary to childhood reflux nephropathy being followed up at our renal unit in St Helier Hospital, Epsom and St Helier University Hospitals NHS Trust, who received a living unrelated donor kidney transplant in January 2024 through the UK national kidney sharing scheme. The HLA mismatch was 1‐1‐1 (A, B and DR loci), and the cytomegalovirus (CMV) status was donor positive, recipient negative (CMV D + R−). Immunosuppressive medication included basiliximab induction, tacrolimus, mycophenolate mofetil (MMF) and prednisolone, with valganciclovir prophylaxis for 90 days. On Day 3 postop, she dropped her haemoglobin (Hb). She was transfused with 3 units of blood, and imaging revealed a large haematoma, which was evacuated in theatre. The Hb stabilised between 82 and 85 g/L, and the graft function was excellent with an eGFR of > 70 mL/min/1.73m^2^. In spite of excellent transplant kidney function, anaemia continued to be an issue during her post‐transplant clinic visits, with adequate iron stores and normal haematinics. We initially attributed the anaemia to MMF. At 4‐weeks post‐transplant, the Hb dropped to 65 g/L with no evidence of active bleeding in or around the transplant kidney on CT angiography, and no evidence of gastrointestinal bleeding (clinically and on endoscopies). The white cell and platelet counts were normal. Haematinics were normal apart from very high ferritin levels (peaked at 4500 *μ*g/L) pretransfusion, with normal C‐reactive protein. She had intermittently raised alanine aminotransferase (ALT), which subsequently normalised. There was no evidence of haemolysis on the blood film.

She was given 2 units of blood and started on erythropoietin injections. Apart from fatigue and lethargy, she had no other systemic symptoms. In view of the abnormal ALT and unexplained anaemia, we suspected CMV (although unlikely whilst on valganciclovir prophylaxis) and Epstein Barr virus (EBV). The EBV and CMV PCRs were negative on several occasions, and the viral hepatitis screen was negative. There was no leukopaenia or thrombocytopaenia during this early period.

The anaemia persisted into March 2024. As she had an early post‐transplant bleed resulting in peritransplant haematoma, we did another CT transplant kidney angiogram including arterial and venous phase images. The scan showed no active bleeding.

We suspected parvovirus infection, but the reticulocyte counts were normal at this stage. The parvovirus B19 IgG and IgM antibodies were negative on 22^nd^ and 25^th^ March 2024. However, the parvovirus B19 DNA PCR came back strongly positive at > 100 billion DNA copies/mL on 25^th^ March 2024 and on 10^th^ April 2024. Retrospective analysis of stored samples detected parvovirus DNA in high titres from a sample from the 1^st^ week of February 2024. Table 1 summarises the parvovirus test results. Neutropaenia was noted in the 1^st^ week of April 2024. Low reticulocyte counts were noted for the first time in mid‐April 2024, whilst severe anaemia preceded this by 6–8 weeks. Peripheral blood films demonstrated red cell anisopoikilocytosis, toxic neutrophils, polychromasia with macrocytes and occasional tear drop cells. The vitamin B12 and folate levels were consistently normal.

**Table 1:** Parvovirus antibody test results and PCR viral load quantification results.

	10/01/2024 (pre transplant, retrospective test)	01/02/2024 (retrospective test)	22/02/2024	25/03/2024	10/04/2024	22/04/2024	07/05/2024	09/2025	02/2026
IgM		Not detected	Not detected	Not detected	Detected				
IgG		Not detected	Not detected	Not detected	Not detected		Detected		
Viral load (copies/mL)	Not detected	13,300,000,000		5,620,000,000		372,000		14,151 IU/mL	1259 IU/mL

She was initially managed with reduction and then cessation of MMF. Due to persistent anaemia and a very high‐grade viraemia, we administered IV immunoglobulin (2 g/kg calculated to ideal body weight, 50‐g total dose), on 13^th^ and 14^th^ April 2024. The haematological indices responded well, with a sustained improvement in Hb level and normalisation of neutrophil counts. In May 2024, she sero‐converted with positive IgG and IgM parvovirus antibodies. The transplant kidney function remained excellent and stable throughout with no significant albuminuria at any stage.

**Figure 1:**
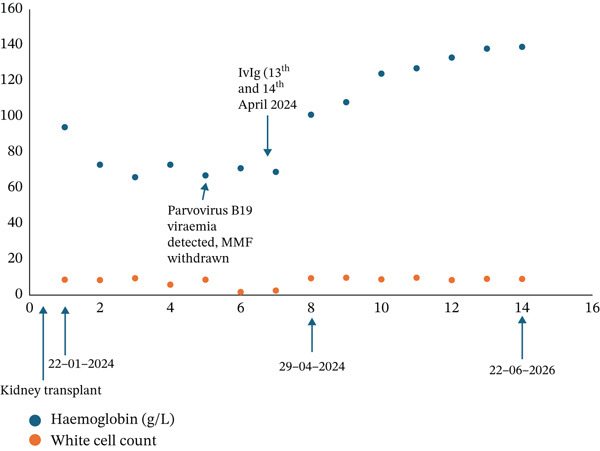
Haemoglobin and white cell count in relation to the detection of parvovirus B19 viraemia, immunosuppression reduction and intravenous immunoglobulin administration MMF = mycophenolate mofetil. IvIg = intravenous immunoglobulin.

Figure 1 shows the haematological parameters from the time of transplantation.

Based on the improved haematological indices and declining parvovirus DNA titres, we re‐introduced MMF (as she was a highly sensitised recipient with 85% HLA antibody calculated reaction frequency [cRF] pretransplant), but there was a rebound increase in parvovirus DNA copies to > 40 billion copies/mL, following which MMF was stopped.

She remains well to date on tacrolimus‐prednisolone immunosuppression with a stable Hb of 115–127 g/L and excellent kidney function, but with an ongoing low‐grade parvovirus viraemia of 50,000–100,000 copies/mL, last tested in early 2025, 14,151 IU/mL in September 2025, with further reductions in viral load when tested in February 2026 (1259 IU/mL) (Table 1). Latest blood test results from June 2026 showed Hb of 132 g/L, normal white cell and platelet counts, ferritin 393 *μ*g/L, creatinine 108*μ*moL/L and eGFR 50 mL/min/1.73m^2^.

## 3. Discussion

Parvovirus B19 causes a mild self‐limiting illness in children (erythema infectiosum) but can cause severe red cell aplasia in immunocompromised hosts, as seen in our patient. Humans are the only host for this virus. The Parvoviridae (name derived from Latin, parvum = small) are subdivided into eight genera including erythroparvovirus B19 (3 genotypes). Parvovirus B19 is a nonenveloped, icosahedral, single‐stranded DNA virus (5600 bp), 23–26 nm in diameter . The virus has a specific tropism for CD36 human erythroid progenitor cells, with viral binding to globoside (P blood group antigen found on red blood cells and their precursors), and, therefore, the predominant clinical presentation is severe anaemia. Globoside antigen is found in reduced numbers on other (nonerythroid) cell types [4].

The anaemia in the early post‐transplant period in our patient was due to peri‐operative bleeding resulting in a large haematoma around the transplant. With the subsequent drop in Hb (in association with a normal reticulocyte count), our focus was to exclude any bleeding from arterial or venous anastomoses of the transplant kidney. MMF is a known cause of anaemia in kidney transplant patients with or without leukopaenia, and we felt that the lack of a reticulocyte response (to a possible bleed) was due to the bone marrow suppressive effect of MMF. Once we excluded peritransplant kidney active bleeding and bleeding from the gastrointestinal tract, we turned our focus into investigating for infective causes of anaemia. Very high ferritin levels supported a systemic inflammatory response to an infection.

Both IgM and IgG antibodies against parvovirus were negative in the early stages of infection in our patient, and the seroconversion occurred late (around 8–10 weeks from onset), highlighting the effect of immunosuppressive therapy on antibody production which could lead to delays in diagnosis if we had relied on them for making a diagnosis.

Our case is a reminder that early whole blood PCR to detect the virus is essential to make a diagnosis even in the absence of supportive serology. One would expect low reticulocyte counts in parvovirus infections, but in our patient the reticulocyte counts were normal initially. This may be due to bone marrow stimulation from high‐dose erythropoietin that she was receiving at that time. The white cell counts remained normal for most of our patient′s clinical course.

The markedly elevated ferritin level and transient liver enzyme abnormalities raise the possibility of a virus‐induced hyperinflammatory state or HLH‐like syndrome; however, formal diagnostic criteria for HLH were not established. The high ferritin levels and transaminitis responded to the falling viral load upon reduction of immunosuppressive medication and intravenous immunoglobulin treatment.

The infection occurred very early in the post‐transplant period which made us wonder if she had a subclinical infection at the time of transplantation, and to test this, we retrospectively analysed a pre‐transplant (Day minus 1) stored blood sample, but it was negative for the virus by PCR, thus excluding this possibility. It is possible that she acquired the virus from her donor, but the donor centre confirmed that donor was completely asymptomatic with no viral illness and no abnormalities in any of the blood tests both pre and postdonation. There was no clear history suggestive of ‘erythema infectiosum’ facial skin rash in either the patient or close family contacts. Therefore, the source of infection in our patient remains unclear.

This case underscores the value of PCR to detect viral nucleic acid for diagnosis of viral infections in immunocompromised hosts as serology may not be reliable, as in our case. Reduction in immunosuppressive therapy (withdrawal of MMF and aiming for lower tacrolimus target levels), coupled with intravenous immunoglobulin therapy, proved effective in managing the severe anaemia secondary to parvovirus‐B19 infection in our patient.

The persistent viraemia > 6 months after diagnosis in our patient suggests a state of chronic infection with parvovirus B19. Unlike in children and young adults with acute infection with parvovirus B19 which is a self‐limiting illness, in immunocompromised hosts, the viraemia can be chronic, which means our patient remains susceptible to worsening viraemia and anaemia during periods of increased immunosuppression [5].

This persistence of viraemia stems from the ability of the virus to reside chronically within early erythroid progenitor cells when T‐cell mediated immunity is suppressed, and this has been documented previously in kidney transplant patients in the context of parvovirus B19 infection with relapsing red cell aplasia [6].

Our plan is to give her further infusions of intravenous immunoglobulin if there is recurrence of severe anaemia associated with worsening parvovirus B19 DNA viraemia.

Although parvovirus B19 infection causing severe anaemia following kidney transplantation is thought to be uncommon, a recent meta‐analysis reported a 1‐year 10.3% pooled estimated incidence rate of parvovirus B19 DNA PCR positivity and a higher 27.4% PCR positivity rate in kidney transplant patients with anaemia [7]. While the choice of induction agent and the degree of immunosuppression will have a bearing on incidence of parvovirus infection, this study highlights that the virus is a common but frequently under‐recognised cause of refractory post‐transplant anaemia.

## 4. Conclusions

The clinicians taking care of kidney transplant patients with severe unexplained anaemia should have a low threshold for testing for parvovirus B19 infection. The serology is not reliable for diagnosis as seroconversion may not happen or may be delayed in an immunocompromised host as shown in our case, leading to a delay in diagnosis. Whole blood DNA PCR is recommended to make a diagnosis when there is clinical suspicion. Chronic persistent or recurrent viraemia can occur in an immunocompromised host. Treatment includes reduction in immunosuppressive medication and administration of intravenous immunoglobulin in selected cases.

## Funding

No funding was received for this manuscript.

## Disclosure

This work was presented in abstract form at the UKKW 2025 (UK Kidney Week) conference.

## Consent

A written consent was obtained from the patient for publication of this case report including blood results and images (where applicable).

## Conflicts of Interest

The authors declare no conflicts of interest.

## Data Availability

The data that support the findings of this study are available from the corresponding author upon reasonable request.
